# Community daytime noise pollution and socioeconomic differences in Chicago, IL

**DOI:** 10.1371/journal.pone.0254762

**Published:** 2021-08-04

**Authors:** Yu-Kai Huang, Uchechi A. Mitchell, Lorraine M. Conroy, Rachael M. Jones

**Affiliations:** 1 Division of Environmental and Occupational Health Sciences, School of Public Health, University of Illinois at Chicago, Chicago, Illinois, United States of America; 2 Division of Community Health Sciences, School of Public Health, University of Illinois at Chicago, Chicago, Illinois, United States of America; 3 Department of Family and Preventive Medicine, School of Medicine, University of Utah, Salt Lake City, Utah, United States of America; SUNY Downstate: SUNY Downstate Health Sciences University, UNITED STATES

## Abstract

Environmental noise may affect hearing and a variety of non-auditory disease processes. There is some evidence that, like other environmental hazards, noise may be differentially distributed across communities based on socioeconomic status. We aimed to a) predict daytime noise pollution levels and b) assess disparities in daytime noise exposure in Chicago, Illinois. We measured 5-minute daytime noise levels (L_eq, 5-min_) at 75 randomly selected sites in Chicago in March, 2019. Geographically-based variables thought to be associated with noise were obtained, and used to fit a noise land-use regression model to estimate the daytime environmental noise level at the centroid of the census blocks. Demographic and socioeconomic data were obtained from the City of Chicago for the 77 community areas, and associations with daytime noise levels were assessed using spatial autoregressive models. Mean sampled noise level (L_eq, 5-min_) was 60.6 dBA. The adjusted R^2^ and root mean square error of the noise land use regression model and the validation model were 0.60 and 4.67 dBA and 0.51 and 5.90 dBA, respectively. Nearly 75% of city blocks and 85% of city communities have predicted daytime noise level higher than 55 dBA. Of the socioeconomic variables explored, only community per capita income was associated with mean community predicted noise levels, and was highest for communities with incomes in the 2^nd^ quartile. Both the noise measurements and land-use regression modeling demonstrate that Chicago has levels of environmental noise likely contributing to the total burden of environmental stressors. Noise is not uniformly distributed across Chicago; it is associated with proximity to roads and public transportation, and is higher among communities with mid-to-low incomes per capita, which highlights how socially and economically disadvantaged communities may be disproportionately impacted by this environmental exposure.

## Introduction

Environmental noise in urban areas has long been recognized as annoying and adversely affecting sleep [1, 2]. Evidence is emerging, however, that environmental noise is associated with a variety of non-auditory disease processes, including: ischemic heart disease [3], hypertension [4], cognitive function [5], and diabetes [6], among others. These conditions are more prevalent among low-income, disadvantaged populations [7–10], which suggests a potential mechanistic role of environmental noise in health disparities. To better elucidate the effect of noise on human health, it is important to improve our understanding of the determinants of noise exposures in urban environments and identify at-risk populations. This is challenging in epidemiologic studies because noise is one of many agents comprising the urban exposome [11], and can be correlated with other exposures [6].

Noise is thought to influence cardiometabolic health through a physiological stress response [12], a response influenced by a variety of social and environmental stressors. Repeated and prolonged exposure to social and environmental stressors activates the sympathetic-adrenal-medulla system and the hypothalamic-pituitary-adrenal axis, triggering the release of primary mediators of the stress response including norepinephrine, epinephrine and cortisol [13]. These mediators then activate secondary responses across key regulatory systems of the body, including the immune, cardiovascular and metabolic systems. Although the physiological stress response is adaptive during short-lived (i.e., acute) stress exposures, repeated and prolonged exposure to environmental stressors can exert a physiological toll on the body that alters normal functioning and leads to the dysregulation of multiple, interconnected physiological systems [14]. Living in urban areas with high levels of environmental noise may contribute to this process of dysregulation.

Structural racism and economic inequities have resulted in residential and economic segregation, such that communities of color and low-income individuals are often relegated to neighborhoods characterized by concentrated poverty, economic disinvestment and political marginalization [15]. Chicago, IL is a large, racially and ethnically diverse city that continues to experience segregation based on race, ethnicity and socioeconomic status. Urban redevelopment initiatives and housing policies in the 1940s through the 1960s contributed to the segregation of African Americans and Latinos to the South and West sides of the city from non-Latino whites in the North and East [16]. The impact of residential segregation has been the disproportionate clustering of adverse health exposures, including noise, in some socially and economically disadvantaged neighborhoods [17, 18].

The objectives of this study were to: a) predict daytime noise pollution levels and b) assess disparities in daytime noise exposure in Chicago, IL, USA. This study aims to better elucidate the association between environmental noise level and socioeconomic status of Chicago’s communities.

## Materials and methods

### Noise sampling

**Fig 1:**
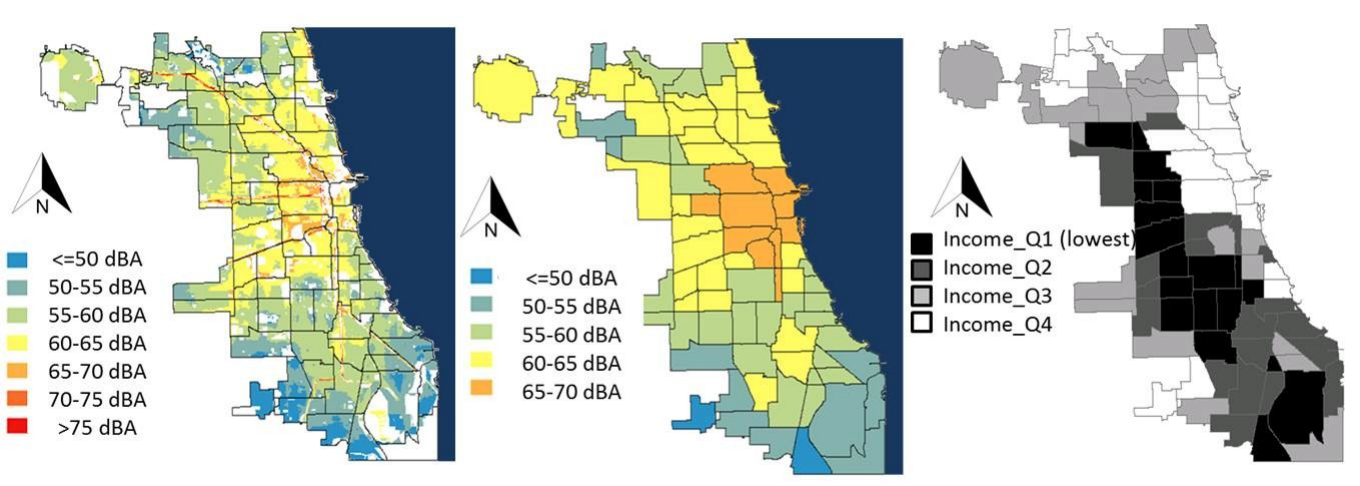
Predicted daytime noise level in the city of Chicago, IL at the census block (left) and community level (middle), compared to community per capita income (right). Black lines denote the 77 communities.

Chicago, IL has an area of 606 km^2^ (Fig 1), and is comprised of 77 community areas and 46,357 census blocks. Seventy-five sites were selected for measurement from contiguous 200 m^2^ tiles using stratified random sampling with proportional allocation on two potential determinants of noise: proximity to airports and greenness. These two determinants were selected because proximity to airports is a distinct determinant of noise, and greenness is used to mitigate noise in urban areas [19]. For some sites, the exact location was moved from the tile centroid due to accessibility. The geolocation of the sites was extracted from Google Maps (Google, Mountain View, CA).

Five-minute average daytime noise levels (L_eq, 5-min_, dBA) were measured at each site using the 3M SoundPro sound level meter (3M, Maplewood, MN) on the fast A-weighted setting. A-weighting is used to adjust the sound pressure levels to reflect the response of the human ear. The sound level meter was calibrated using the AcoustiCal AC-300 sound level calibrator at a sound pressure level of 114 dB before and after the sampling periods. The value of L_eq, 5-min_, dBA was automatically calculated by the sound level meter. The sound level meter was held at a height of 1.5 m and a windscreen was used on the microphone (Type 4936, Brüel & Kjær, Nærum, Denmark). Noise measurements were made in the “non-rush hour” period between 10 AM and 4 PM in March 2019 (3/8/2019-3/21/2019). Noise sampling in urban areas has been previously performed using this method, and L_eq, 5-min_ measurements have been found to be stable over time [20, 21].

### Geographically-based variables

Public sources of digitized data were used to identify and calculate a number of geographically-based variables at each of the 75 sampling sites and the centroids of the 46,375 census blocks in Chicago. All layer attribution extraction and variable generation were performed using ArcGIS 10.5 (Esri, Redlands, CA). For each variable, the anticipated association with environmental noise (positive/negative/unknown) was specified a priori, based on published literature. The variables can be grouped into four major categories:

1. *Land use*. Residential, commercial and industrial zoning was calculated based on 2018 Zoning Districts from the City of Chicago data portal (https://data.cityofchicago.org).
2. *Aviation traffic*. Chicago has two airports–Chicago O’Hare International Airport and Chicago Midway International Airport. The airport locations were extracted from Google Maps. Noise contours around the airports were based on the Airport Noise Management System reports [22, 23], which depicted the area with day-night average sound levels higher than 65 dB based on Federal Aviation Administration’s Integrated Noise Model.
3. *Ground traffic*. Ground traffic variables included distance to nearest and total length of surrounding streets, major roads, primary roads, Chicago Transit Authority (CTA) train tracks, CTA bus routes and Metra train tracks. Primary road information was obtained from the 2016 data from US Census Bureau, Department of Commerce (data.gov), and the rest of the variables were obtained from data on the City of Chicago data portal.
4. *Natural environment*. Natural environment variables included distance to Lake Michigan and Normalized Difference Vegetation Index (NDVI). NDVI was calculated from the public raster Landsat-8 Satellite image obtained from USGS Earth Explorer (https://earthexplorer.usgs.gov). NDVI ranges from -1 to 1, where positive values indicate vegetated area and negative values indicate non-vegetated area, such as water surface or barren land [24].

The complete list of variables is available in S1 Table in S1 File, and some are depicted in S1 Fig in S1 File. Buffer radii of 100, 200, 300, 500 and 1000 meters were used to define area variables, and are noted as subscripts in the variables. For area variables doughnut-shaped regions were also defined: NDVI_500-100_, for example, is the difference between NDVI_500_ and NDVI_100_. When multiple years of data were available, the most recent was used.

### Descriptive analysis

Descriptive analysis for noise level measurements and certain geographically-based variables was conducted, and included tabulation of the mean, standard deviation and selected percentiles.

### Land-use regression modelling

A land-use regression (LUR) model was built to predict the spatial pattern of noise in Chicago, IL. The measured noise level (L_eq, 5min_) was the dependent variable and the geographically-based variables were the independent variables. Prior to model fitting, the 75 sampling sites were randomly divided into a training set (n = 38) and a validation set (n = 37).

The supervised stepwise method was used to build the LUR model with the training set [25]. Initially, LUR models were fitted for one geographically-based variable at a time. The variable that gave the highest R^2^ in the single-variable models was selected as the first variable for inclusion in the multivariate LUR model. Individual variables were then added, and retained in the multivariate LUR model when the adjusted R^2^ improvement was greater than 0.01. Variables added included interaction and exponential terms. During the stepwise selection process, we only retained variables in the model that met the following criteria:

1. The regression coefficient direction of each independent variable was the same as our a priori determination of the direction of effect.
2. The p-value of each independent variable was smaller than 0.2.
3. The variance inflation factor (VIF) of each independent variable was smaller than 4 [26].

To validate the model, the final fitted multivariate LUR model was used to predict noise levels at the 37 sites in the validation set. Model performance was determined by comparing predicted and measured noise levels at these 37 sites using the adjusted R^2^ and root mean square error (RMSE).

### Socioeconomic analysis

The final LUR model was used to predict noise levels at the geo-centroid of the 46,357 census blocks in Chicago, IL. The mean noise level, $\bar{N}$ (dBA), for each of the 77 Chicago community areas was calculated using the predicted noise level *N_i_* for each of the *n* census blocks within each community:

$$\bar{N}={log}^{\left[\frac{1}{n}\times 10^{\left(\frac{N_{1}}{10}\right)}+\frac{1}{n}\times 10^{\left(\frac{N_{2}}{10}\right)}+\cdots +\frac{1}{n}\times 10^{\left(\frac{N_{n}}{10}\right)}\right]}$$

Demographic and socioeconomic data for the 77 community areas were obtained from the 2008–2012 and 2012–2016 American Community Survey through the City of Chicago data portal. Ten variables were selected for analysis, including 1) population density, 2) percent commuters, 3) White race, 4) Hispanic ethnicity, 5) Black race, 6) Asian race, 7) population without a high school diploma, 8) population unemployed, 9) per capita income and 10) economic hardship index. The hardship index is an indicator of economic conditions based on the following: the percent of the population living below the poverty level, unemployed, without a high school diploma, less than 18 years old and aged 65 years or older, per capita income, and crowding level (percentage of housing units with more than one occupant per room) [27, 28]. Percent communters were included because it may reflect an abundance of public transit options, or lack of financial resources for private transportation.

Moran’s I test, using queen’s case nearest neighbors, indicated mean daytime noise in the 77 communities was spatially autocorrelated, with I = 0.59 given a possible range of -0.83 to 1.03 [29]. As a result, a spatial autoregressive model (SAR) was implemented using the *lagsarlm* function of the ‘spdep’ package in R to explore the association between environmental noise and socioeconomic variables [30]. Since previous studies suggested that socioeconomic variables may be non-linearly associated with noise [17], these variables were divided into quartiles for regression modeling. Locally weighted smoothing (LOESS) was used to graphically explore the relationships between quartiles of the socioeconomic variables with predicted community noise levels. As with the LUR model, a supervised stepwise method was used. First, each socioeconomic variable was separately included in the model. Then, additional variables were included only when at least one of the quartile dummy variables had p-value < 0.1. Interim results were inspected to evaluate whether additional variables influenced that coefficient of other variables. Cook’s D and DFFITS were used to identify and assess the influence of potential outliers in the regression model.

## Results

### Noise measurements

The mean measured L_eq-5min_ is 60.6 dBA, with a range of 48.1–82.0 dBA (Table 1); the noise sampling sites are shown in S2 Fig in S1 File. The distributions of the geographically-based variables included in the final LUR model at the 75 noise sampling sites are fairly similar to the distributions for the 46,357 census blocks in Chicago, IL (Table 1). Only the range of values for NDVI_100_ and NDVI_500_ are noticeably smaller than those for the city. Overall, the characteristics of the geographically-based variables at the noise sampling sites were similar to those of the city, though 5,222 of the 46,357 (11.3%) census blocks in the city require predictions outside of the range of collected data (S2 Fig in S1 File).

**Table 1:** Measured noise levels (L_eq-5min_) and geographically-based variables included in the final land use regression model at the noise sampling sites (n = 75) and at the centroids of the census blocks in Chicago, IL (n = 46,375).

Variable	Unit	Noise Sampling Sites			Chicago		
		Mean	SD	0, 50, 100%tiles	Mean	SD	0, 50,100%tiles
L_eq-5min_	dBA	60.6	7.9	48.1, 60.8, 82.0			
D_CTA_	m	2065	2239	4, 1393, 10720	1999	1992	0, 1258, 11419
PRD_100_	m	12	76	0, 0, 577	37	209	0, 0, 2679
NDVI_100_		0.32	0.15	0.05, 0.31, 0.70	0.35	0.12	-0.61, 0.36, 0.90
NDVI_500_		0.33	0.12	-0.03, 0.34, 0.81	0.35	0.09	-0.27, 0.35, 0.87
NDVI_500_-_100_		-0.00	0.11	-0.27, 0.00, 0.28	0.00	0.08	-0.55, 0.00, 0.53

D_CTA_: the distance to the nearest CTA train track. PRD_100_: the total length of primary road within 100 meters. NDVI_100_: the mean NDVI within 100 meters. NDVI_500_: the mean NDVI within 500 meters. NDVI_500-100_: NDVI500 –NDVI_100_

### Daytime noise LUR model and predictions

Table 2 shows the final noise LUR model built on the training set (n = 38) to predict the daytime L_eq-5min_, which included four independent geographically-based variables. Model performance was reasonably good, with adjusted R^2^ = 0.60 for the training set and adjusted R^2^ = 0.51 for the validation set. The regression diagnostics showed a slight heteroscedasticity, but it was not severe (S3 Fig in S1 File). No autoregressive correlation was found through the Moran’s I testing (Moran’s I: 6.09, p < 0.01). Sensitivity analysis verified that the presence of surface water, which has low values of NDVI, did not meaningfully impact the LUR model (S2 Table in S1 File).

**Table 2:** Fitted land use regression model predicting daytime noise (L_eq-5min_, dBA) in Chicago, IL.

Model					Training Set		Validation Set	
Variables	β	SE	p-value	VIF	adj-R^2^	RMSE (dBA)	adj-R^2^	RMSE (dBA)
Intercept	71.95	2.69	< .001		0.60	4.67	0.51	5.90
D_CTA_	-1.69×10^−3^	4.38×10^−4^	< .001	1.19				
NDVI_100_	-29.87	7.36	< .001	1.79				
PRD_100_	2.72×10^−2^	8.46×10^−3^	0.002	1.35				
NDVI_500-100_	-24.50	10.29	0.023	2.08				

D_CTA_: the distance to the nearest CTA train track. PRD_100_: the total length of primary road within 100 meters. NDVI_100_: the mean NDVI within 100 meters. NDVI_500_: the mean NDVI within 500 meters. NDVI_500-100_: NDVI500 –NDVI_100_. RMSE: Root-mean square error. VIF: Variance inflation factor.

The daytime noise level predicted by the LUR model in each of 41,135 census blocks in Chicago that did not require prediction outside the range is shown in Fig 1. The areas with higher daytime noise levels are noticeably close to interstate highways and major arterials (along the lakeshore on the north side), and downtown Chicago. Predicted noise levels had mean 58.2 dBA, median 58.5 dBA, and range 41.5–82.5 dBA. Three-fourths (32,078 of 41,135) of the census blocks had daytime noise levels greater than 55 dBA, the WHO guideline for preventing annoyance [31].

### Association between noise and socioeconomic conditions

**Fig 2:**
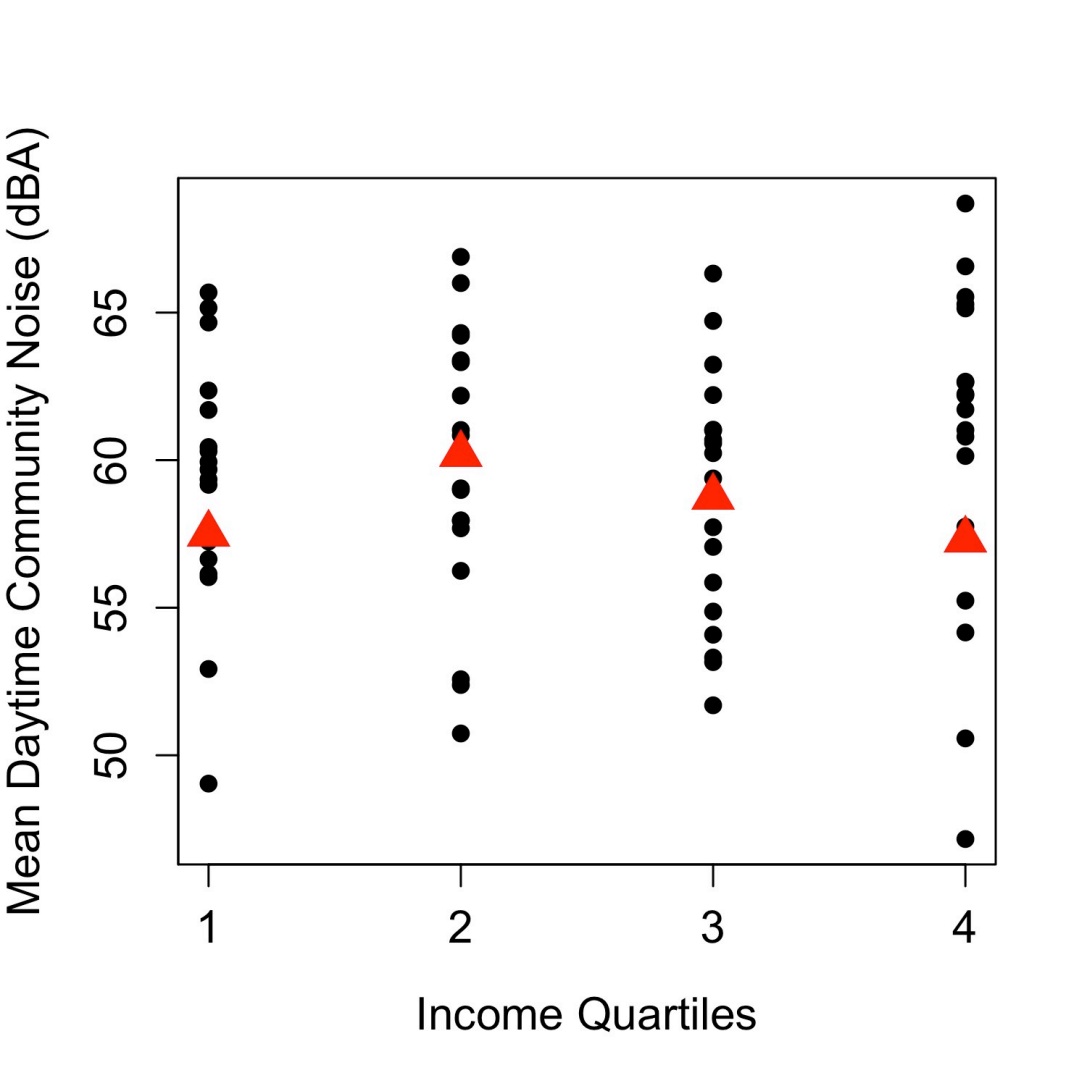
Association between quartiles of community per capita income and mean community daytime noise.

Daytime predicted noise levels for each census block were averaged to estimate the daytime average noise level in the 77 Chicago communities (Fig 1), and are shown in Table 3 with community socioeconomic characteristics. Of the ten socioeconomic variables, only community income per capita was statistically significantly associated with noise (Fig 2, Table 4). In particular, the second quartile of per capita income ($15,800-$21,300) was associated with increased community daytime noise. In previous work, other investigators had adjusted for population and/or population density [17], but the addition of community population density quartiles did not affect the association between per capita income and community daytime noise, nor improve the model (Likelihood ratio 1.73, p = 0.63). The spatial distribution of community areas in the second quartile per capita income overlaps with the location of interstate highways, particularly among those communities on the south and west sides of Chicago (Fig 1). Community area racial and ethnic demographics were not associated with community daytime noise in univariate models (S3 Table in S1 File).

**Table 3:** Predicted daytime noise level and selected socioeconomic variables among 77 Chicago community areas.

Variables	Mean	Q1	Median	Q3	Range
Noise level (dBA)	59.5	57.1	60.1	62.4	47.2–68.7
Population density (1000 person/mile^2^)	17.9	6.0	11.3	18.1	0.9–139.4
% Commuter	41.7	34.4	41.7	48.5	20.9–66.3
% White	27.9	3.4	15.0	47.2	0.4–88.7
% Hispanic	26.1	3.6	12.5	48.1	0.0–92.6
% Black	38.3	2.8	13.7	87.8	0.5–99.1
% Asian	6.0	0.4	1.9	8.3	0.0–75.2
% no high school diploma	20.3	11.8	18.5	26.6	2.5–54.8
% unemployed	15.4	9.2	13.9	20	4.7–35.9
Income per capita (1,000 USD)	25.6	15.8	21.3	28.9	8.2–88.7
Hardship Index	50	25	50	74	1–98

**Table 4:** Spatial auto-regression model for the relationship between community noise level and per capita income quartiles.

Variables	β	SE	p-value	Rho	p-value
Intercept	8.51	3.59	0.018	0.8491	< 0.001
Income Q2	2.03	0.91	0.024		
Income Q3	0.93	0.87	0.287		
Income Q4	0.20	0.94	0.832		

## Discussion

The noise sampling strategy was able to select sites that were representative of potential determinants of noise in Chicago (Table 1), leaving few census blocks outside the range of the observed data. The LUR model performed well with respect to the training and validation data sets, as indicated by the adjusted R^2^ and RMSE (Table 2); similar to the performance of other LUR models of noise in urban areas [32–34].

Independent variables included in the final LUR model–length of primary roads, proximity to public transportation, and vegetation–are all plausible determinants of environmental noise in urban environments (Table 2). Monitoring studies, for example, have found noise levels to be higher near freeways [35] and train tracks [36, 37] than at more distant locations. The highest single measurement in this study, 82 dBA, was collected near a busy elevated CTA train station, in an area of high commercial and traffic activity. Phan and Jones [38] found that noise levels were higher on elevated CTA trains and platforms than on ground-level trains and platforms, and high commercial and traffic activity have been associated with elevated noise levels [39, 40]. Areas with more vegetation in Chicago, as in many urban areas, are less densely built, and have been associated with lower levels of environmental noise [41–43].

The daytime L_eq-5min_ noise levels measured in this study (mean 60.6 dBA, range 48.1–82 dBA, Table 1) are similar to those measured at other sites in Chicago in 2006 and 2007, which had mean of 59.3 dBA (range: 48.1–72.1 dBA) and 60.4 dBA (range: 51.3–73.4 dBA), respectively [20]. Further, L_eq-5min_ noise levels measured at the same sites in 2006 and 2007 were highly correlated (r = 0.84) [20]. While noise sampling sites in this study differed from those in Allen et al., the data suggest daytime noise levels have not changed substantially in the past decade [20]. Daytime noise levels in Chicago are similar to those in other American cities, including Boston, MA [44] and New York City, NY [45].

The predicted daytime noise levels at 75% of the census blocks and 83% of communities in Chicago are higher than 55 dBA, a guideline value from the WHO set to prevent serious annoyance [31]. This means that the majority of Chicago residents experience average daytime outdoor noise levels associated with annoyance and able to activate a physiological response to stress associated with diverse adverse health outcomes. Owing to the sampling strategy used in this study, it is likely that Chicago residents experience higher transient noise levels, such as from an overhead airplane, passing trucks or construction activities.

Of the ten socioeconomic variables explored, community daytime noise level was only statistically significantly associated with community per capita income, with higher daytime noise levels associated with the second income quartile relative to the first quartile (Fig 2, Table 4). Community per capita income is strongly associated with race and ethnicity in Chicago, and the spatial distribution of communities with low per capita income (Fig 1) reflects well known patterns of residential segregation [16]. Inspection of Fig 1 suggests that the elevated noise levels in communities in the second income quartile is likely the result of interstate highways that pass through these communities, and the mean total highway length is highest in this quartile, with 8714 m, compared to 5947 m, 4629 m and 5841 m in the first, third and fourth quartiles, respectively (S4 Table and S4 Fig in S1 File). Although we found not association between community daytime noise and racial and ethnic demographics in this study, historically, the placement and construction of highways were racially and economically motivated [46] and contributed to “white flight” to the suburbs [47]. This effectively weakened the local tax base in major cities like Chicago, furthering residential segregation and economic disinvestment. As a result, lower income communities are primarily located near highways and traffic-related exposures [48] but the communities with lowest income are physically isolated from infrastructure that facilitates transportation and commercial activity, increasing disparity and deprivation. Thus, communities in the first quartile of per capita income in Chicago are less likely to include interstate highways than communities in the second quartile of per capital income. In contrast, communities in the highest quartile of per capita income in Chicago have increased CTA service (S4 Table in S1 File).

The socioeconomic variables explored in this study were from the American Community Survey, which is known to be affected by uncertainty that is not uniformly distributed across census blocks. For example, Folch et al. [49] identified imprecision in median household income to be higher in lower-income areas and in urban areas. This issue introduces uncertainty and error into the analysis of the relationship between noise and socioeconomic variables, but the impact is minimized by use of quartiles for socioeconomic variables.

Application of the LUR model to epidemiologic and socioeconomic disparities may be limited by the noise metric used in sampling, daytime L_eq-5min_. While short-term daytime noise levels have been found to be highly correlated with other metrics of noise levels and times of days [20, 45], other noise metrics, such as L_n_ (night-average sound level) or L_den_ (day-night average sound level) that capture nighttime noise, may be more biologically relevant to adverse health outcomes or disparities. For instance, Casey et al. [17] found statistically significant differences in nighttime noise levels among urban block groups with more black residents and residents living in poverty experiencing greater nighttime noise.

In this study, all noise measurements were collected in the spring on weekdays, though other work suggests environmental noise levels may vary seasonally and by day of week [44], such as may arise because of changes in traffic levels. With respect to human exposure, seasonal variation may be driven by human behavior (time indoors and outdoors) and effects of socioeconomic conditions (access to air conditioners rather than open windows), both of which impact time spent outdoors and indoor noise exposure. When people spend more time indoors with closed windows, exposure to outdoor noise may decrease while exposure to indoor noise increases. Future research should address these sources of variation, and capture human exposure directly through personal noise dosimetry over 24-hour periods. This will allow for more robust assessment of noise exposure and exposure disparities that could contribute to adverse health impacts.

## Conclusions

Both the noise measurements and LUR modeling demonstrate that Chicago has high levels of environmental noise, sufficient to cause annoyance and likely contributing to the total burden of environmental stressors. Noise is not uniformly distributed across the city; it is associated with proximity to roads and public transportation, and is higher among communities with mid-to-low incomes per capita, which highlights how socially and economically disadvantaged communities may be disproportionately impacted by this environmental exposure.

## Supporting information

S1 File (DOCX)

## References

[1] Guski R, Schreckenberg D, Schuemer R. WHO Environmental Noise Guidelines for the European Region: A Systematic Review on Environmental Noise and Annoyance. Int J Environ Res Public Health. 2017;14(12):1539. doi: 10.3390/ijerph14121539 29292769 PMC5750957

[2] Basner M, McGuire S. WHO Environmental Noise Guidelines for the European Region: A Systematic Review on Environmental Noise and Effects on Sleep. Int J Environ Res Public Health. 2018;15(3):519. 10.3390/ijerph15030519 PMC5877064 29538344

[3] Ev Kempen, Casas M, Pershagen G, Foraster M. WHO Environmental Noise Guidelines for the European Region: A Systematic Review on Environmental Noise and Cardiovascular and Metabolic Effects: A Summary. Int J Environ Res Public Health. 2018;15(2):379. 10.3390/ijerph15020379 PMC5858448 29470452

[4] van Kempen E, Babisch W. The quantitative relationship between road traffic noise and hypertension: a meta-analysis. Journal of hypertension. 2012;30(6):1075–86. doi: 10.1097/HJH.0b013e328352ac54 22473017

[5] Tzivian L, Dlugaj M, Winkler A, Weinmayr G, Hennig F, Fuks KB, et al. Long-Term Air Pollution and Traffic Noise Exposures and Mild Cognitive Impairment in Older Adults: A Cross-Sectional Analysis of the Heinz Nixdorf Recall Study. Environ Health Perspect. 2016;124(9):1361–8. doi: 10.1289/ehp.1509824 26863687 PMC5010410

[6] Klompmaker J, Janssen N, Bloemsma L, Gehring U, Wijga A, van den Brink C, et al. Associations of combined exposures to surrounding green, air pollution, and road traffic noise with cardiometabolic diseases. Envrionmental Health Perspectives. 2019;127(8):87003. doi: 10.1289/EHP3857 31393793 PMC6792364

[7] Rosengren A, Smyth A, Rangarajan S, Ramasundarahettige C, Bangdiwala SI, AlHabib KF, et al. Socioeconomic status and risk of cardiovascular disease in 20 low-income, middle-income, and high-income countries: the Prospective Urban Rural Epidemiologic (PURE) study. The Lancet Global health. 2019;7(6):e748–e60. doi: 10.1016/S2214-109X(19)30045-2 31028013

[8] Schultz WM, Kelli HM, Lisko JC, Varghese T, Shen J, Sandesara P, et al. Socioeconomic Status and Cardiovascular Outcomes: Challenges and Interventions. Circulation. 2018;137(20):2166–78. doi: 10.1161/CIRCULATIONAHA.117.029652 29760227 PMC5958918

[9] Gaskin DJ, Thorpe RJ Jr., McGinty EE, Bower K, Rohde C, Young JH, et al. Disparities in diabetes: the nexus of race, poverty, and place. Am J Public Health. 2014;104(11):2147–55. doi: 10.2105/AJPH.2013.301420 24228660 PMC4021012

[10] Rabi DM, Edwards AL, Southern DA, Svenson LW, Sargious PM, Norton P, et al. Association of socio-economic status with diabetes prevalence and utilization of diabetes care services. BMC Health Serv Res. 2006;6:124–. doi: 10.1186/1472-6963-6-124 17018153 PMC1618393

[11] Robinson O, Tamayo I, de Castro M, Valentin A, Giorgis-Allemand L, Hjertager Krog N, et al. The Urban Exposome during Pregnancy and Its Socioeconomic Determinants. Environmental health perspectives. 2018;126(7):077005–. doi: 10.1289/EHP2862 30024382 PMC6108870

[12] Munzel T, Sorensen M, Gori T, Schmidt FP, Rao X, Brook J, et al. Environmental stressors and cardio-metabolic disease: part I-epidemiologic evidence supporting a role for noise and air pollution and effects of mitigation strategies. European heart journal. 2017;38(8):550–6. doi: 10.1093/eurheartj/ehw269 27460892

[13] McEwen BS. Protective and damaging effects of stress mediators: central role of the brain. Dialogues Clin Neurosci. 2006;8(4):367–81. doi: 10.31887/DCNS.2006.8.4/bmcewen 17290796 PMC3181832

[14] McEwen BS. Stress, adaptation, and disease. Allostasis and allostatic load. Annals of the New York Academy of Sciences. 1998;840:33–44. doi: 10.1111/j.1749-6632.1998.tb09546.x 9629234

[15] Massey DS. American Apartheid: Segregation and the Making of the Underclass. American Journal of Sociology. 1990;96(2):329–57.

[16] Hirsch AR. Making the Second Ghetto: Race and Housing in Chicago 1940–1960. 1988.

[17] Casey JA, Morello-Frosch R, Mennitt DJ, Fristrup K, Ogburn EL, James P. Race/Ethnicity, Socioeconomic Status, Residential Segregation, and Spatial Variation in Noise Exposure in the Contiguous United States. Environ Health Perspect. 2017;125(7):077017. doi: 10.1289/EHP898 28749369 PMC5744659

[18] Downey L. US Metropolitan-area variation in environmental inequaility outcomes. Urban Stud. 2007;44(5–6):953–77. doi: 10.1080/00420980701256013 21909171 PMC3169206

[19] Watanabe T, Yamada S. Sound attenuation through absorption by vegetation 1996. 175–82 p.

[20] Allen RW, Davies H, Cohen MA, Mallach G, Kaufman JD, Adar SD. The spatial relationship between traffic-generated air pollution and noise in 2 US cities. Environmental research. 2009;109(3):334–42. doi: 10.1016/j.envres.2008.12.006 19193368 PMC3071639

[21] Davies HW, Vlaanderen JJ, Henderson SB, Brauer M. Correlation between co-exposures to noise and air pollution from traffic sources. Occupational and environmental medicine. 2009;66(5):347–50. doi: 10.1136/oem.2008.041764 19017692

[22] Chicago Department of Aviation. The Chicago Department of Aviation’s Airport Noise Management System (Midway) [Available from: http://www.flychicago.com/community/MDWnoise/ANMS/pages/default.aspx.

[23] Chicago Department of Aviation. The Chicago Department of Aviation’s Airport Noise Management System (O’Hare) [Available from: http://www.flychicago.com/community/ORDnoise/ANMS/pages/default.aspx.

[24] Yuan F, Bauer ME. Comparison of impervious surface area and normalized difference vegetation index as indicators of surface urban heat island effects in Landsat imagery. Remote Sensing of Environment. 2007;106(3):375–86.

[25] Brunekreef B. study manual—European Study of Cohorts for Air Pollution Effects 2008 [Available from: http://www.escapeproject.eu/manuals/ESCAPE-Study-manual_x007E_final.pdf.

[26] Amini H, Taghavi-Shahri SM, Henderson SB, Naddafi K, Nabizadeh R, Yunesian M. Land use regression models to estimate the annual and seasonal spatial variability of sulfur dioxide and particulate matter in Tehran, Iran. Science of The Total Environment. 2014;488–489:343–53. doi: 10.1016/j.scitotenv.2014.04.106 24836390

[27] Nathan RP, Adams C. Understanding Central City Hardship. Political Science Quarterly. 1976;91(1):47–62.

[28] UIC Great Cities Institute. Fact Sheet #2: Chicago Community Area Economic Hardship Index 2016 [Available from: https://greatcities.uic.edu/wp-content/uploads/2016/07/GCI-Hardship-Index-Fact-SheetV2.pdf.

[29] Brunsdpn C, Comber L. An Introduction to R for Spatial Analysis and Mapping 2015.

[30] Ord K. Estimation Methods for Models of Spatial Interaction. Journal of the American Statistical Association. 1975;70(349):120–6.

[31] Berglund B, Lindvall T, Schwela DH, Organization WH. Guidelines for community noise. 1999.

[32] Aguilera I, Foraster M, Basagana X, Corradi E, Deltell A, Morelli X, et al. Application of land use regression modelling to assess the spatial distribution of road traffic noise in three European cities. J Expo Sci Environ Epidemiol. 2015;25(1):97–105. doi: 10.1038/jes.2014.61 25227731

[33] Fallah-Shorshani M, Minet L, Liu R, Plante C, Goudreau S, Oiamo T, et al. Capturing the spatial variability of noise levels based on a short-term monitoring campaign and comparing noise surfaces against personal exposures collected through a panel study. Environ Res. 2018;167:662–72. doi: 10.1016/j.envres.2018.08.021 30241005

[34] Harouvi O, Ben-Elia E, Factor R, de Hoogh K, Kloog I. Noise estimation model development using high-resolution transportation and land use regression. J Expo Sci Environ Epidemiol. 2018;28(6):559–67. doi: 10.1038/s41370-018-0035-z 29789670

[35] Shu S, Yang P, Zhu Y. Correlation of noise levels and particulate matter concentrations near two major freeways in Los Angeles, California. Environmental Pollution. 2014;193:130–7. doi: 10.1016/j.envpol.2014.06.025 25016466

[36] Zannin PHT, Engel MS, Fiedler PEK, Bunn F. Characterization of environmental noise based on noise measurements, noise mapping and interviews: A case study at a university campus in Brazil. Cities. 2013;31:317–27.

[37] Bunn F, Zannin PHT. Assessment of railway noise in an urban setting. Applied Acoustics. 2016;104:16–23.

[38] Phan LT, Jones RM. Chicago transit authority train noise exposure. J Occup Environ Hyg. 2017;14(6):D86–d91. doi: 10.1080/15459624.2017.1285490 28278069

[39] King G, Roland-Mieszkowski M, Jason T, Rainham DG. Noise levels associated with urban land use. Journal of urban health: bulletin of the New York Academy of Medicine. 2012;89(6):1017–30. doi: 10.1007/s11524-012-9721-7 22707308 PMC3531357

[40] Ko JH, Chang SI, Kim M, Holt JB, Seong JC. Transportation noise and exposed population of an urban area in the Republic of Korea. Environ Int. 2011;37(2):328–34. doi: 10.1016/j.envint.2010.10.001 21056472

[41] Margaritis E, Kang J. Relationship between green space-related morphology and noise pollution. Ecological Indicators. 2017;72:921–33.

[42] Sakieh Y, Jaafari S, Ahmadi M, Danekar A. Green and calm: Modeling the relationships between noise pollution propagation and spatial patterns of urban structures and green covers. Urban Forestry & Urban Greening. 2017;24:195–211.

[43] Han X, Huang X, Liang H, Ma S, Gong J. Analysis of the relationships between environmental noise and urban morphology. Environmental Pollution. 2018;233:755–63. doi: 10.1016/j.envpol.2017.10.126 29127933

[44] Walker ED, Hart JE, Koutrakis P, Cavallari JM, VoPham T, Luna M, et al. Spatial and temporal determinants of A-weighted and frequency specific sound levels—An elastic net approach. Environmental Research. 2017;159:491–9. doi: 10.1016/j.envres.2017.08.034 28865401 PMC5903552

[45] Kheirbek I, Ito K, Neitzel R, Kim J, Johnson S, Ross Z, et al. Spatial variation in environmental noise and air pollution in New York City. Journal of urban health: bulletin of the New York Academy of Medicine. 2014;91(3):415–31. doi: 10.1007/s11524-013-9857-0 24488652 PMC4074330

[46] Connerly CE. From Racial Zoning to Community Empowerment: The Interstate Highway System and the African American Community in Birmingham, Alabama. Journal of Planning Education and Research. 2002;22(2):99–114.

[47] Massey DS, Denton NA. American Apartheid: Segregation and the Making of the Underclass. Cambridge, MA: Harvard University Press; 1993.

[48] Boehmer TK, Foster SL, Henry JR, Woghiren-Akinnifesi EL, Yip FY. Residential proximity to major highways—United States, 2010. MMWR Supplement. 2013;62(3):46–50. 24264489

[49] Folch DC, Arribas-Bel D, Koschinsky J, Spielman SE. Spatial variation in the quality of American Community Survey Estimates. Demography. 2016;53(5):1535–54. doi: 10.1007/s13524-016-0499-1 27541024

