# Supplementary material for: Community daytime noise pollution and socioeconomic differences in Chicago, IL
Source: PLoS One. 2021 Aug 4;16(8):e0254762. doi: 10.1371/journal.pone.0254762 (PMC8336802; doi:10.1371/journal.pone.0254762)

**Communities’ daytime noise pollution and socioeconomic difference in Chicago, IL**

**Supplemental Materials**

**Contents**

**S1 Table.** List of geographically-based variables…………………………………………2

**S1 Figure.** Geographically-based variables in Chicago…………………………………..4

**S2 Figure.** Noise sampling sites (black triangles) and areas where the LUR model will predict outside the range of geographically-based variables (D_CTA_, PRD_100_, NDVI_100_, and NDVI_500-100_) represented by the noise sampling sites (white)……………………………..6

**S3 Figure.** Regression diagnosis and the predicted noise levels for training and validation set………………………………………………………………………………………….6

**S2 Table.** Fitted land use regression Model predicting noise (Leq_-5min_) in Chicago: Sensitivity analysis excluding two locations close to Lake Michigan…………………….7

**S3 Table.** Univariate spatial auto regression models of the association between community area daytime noise level and racial and ethnic demographics………………..7

**S4 Table.** Chicago Transit Authority (CTA) train track and highway length by income quartile in Chicago community areas…………..………………………………………….7

**S4 Figure.** Distribution of mean total Chicago Transit Authority (CTA) train track length (left) and highway length (right) by income quartile in Chicago community areas………8

**S1 Table. List of geographically-based variables**

| Category | Variable | Description | Unit | Source | Direction of effect |
| --- | --- | --- | --- | --- | --- |
| Land use | RES | Total residential zoning area within xm buffer | meter^2^ | Chicago data portal | unknown |
|  | COM | Total commercial zoning area within xm buffer | meter^2^ | Chicago data portal | positive |
|  | IND | Total industrial zoning area within xm buffer | meter^2^ | Chicago data portal | positive |
| Aviation traffic | D_airport_ | Distance to the nearest airport (O’Hare / Midway) | meter | Google Maps | negative |
|  | D_contour_ | Distance to the nearest noise contour (O’Hare / Midway) | meter | Airport Noise Management System reports | negative |
|  | CONTOUR | Total noise contour area within xm buffer | meter^2^ | Airport Noise Management System reports | positive |
| Ground Traffic | D_PRD_ | Distance to the nearest primary road | meter | US Census Bureau, Department of Commerce | negative |
|  | D_MST_ | Distance to the nearest major street | meter | Chicago data portal | negative |
|  | D_ST_ | Distance to the nearest street | meter | Chicago data portal | negative |
|  | D_CTA_ | Distance to the CTA train track | meter | Chicago data portal | negative |
|  | D_BUS_ | Distance to the nearest CTA bus route | meter | Chicago data portal | negative |
|  | D_Metra_ | Distance to the nearest Metra train track | meter | Chicago data portal | negative |
|  | PRD | Total length of primary road within xm buffer | meter^2^ | US Census Bureau, Department of Commerce | positive |
|  | MST | Total length of major street within xm buffer | meter^2^ | Chicago data portal | positive |
|  | ST | Total length of street within xm buffer | meter^2^ | Chicago data portal | positive |
|  | CTA | Total length of CTA train track within xm buffer | meter^2^ | Chicago data portal | positive |
|  | BUS | Total length of CTA bus route within xm buffer | meter^2^ | Chicago data portal | positive |
|  | Metra | Total length of Metra train track within xm buffer | meter^2^ | Chicago data portal | positive |
| Natural environment | D_Lake_ | Distance to the lake (Lake Michigan) | meter | Google Maps | unknown |
|  | NDVI | Mean Normalized Difference Vegetation Index (NDVI) within xm buffer | - | Landsat-8, USGS Explorer | negative |
| xm: five buffer radii used in this study were 100/200/300/500/1000 meter | | | | | |

**S1 Figure. Geographically-based variables in Chicago. Some of these figures utilized data and information from the U.S. Geological Survey.**

a) Land use (zoning) b) Airport noise contours


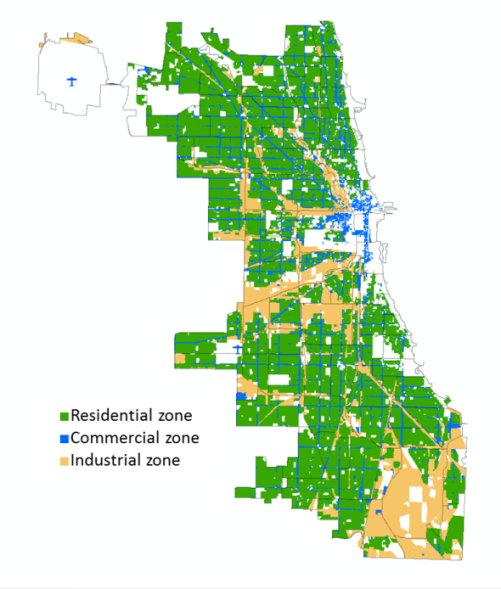

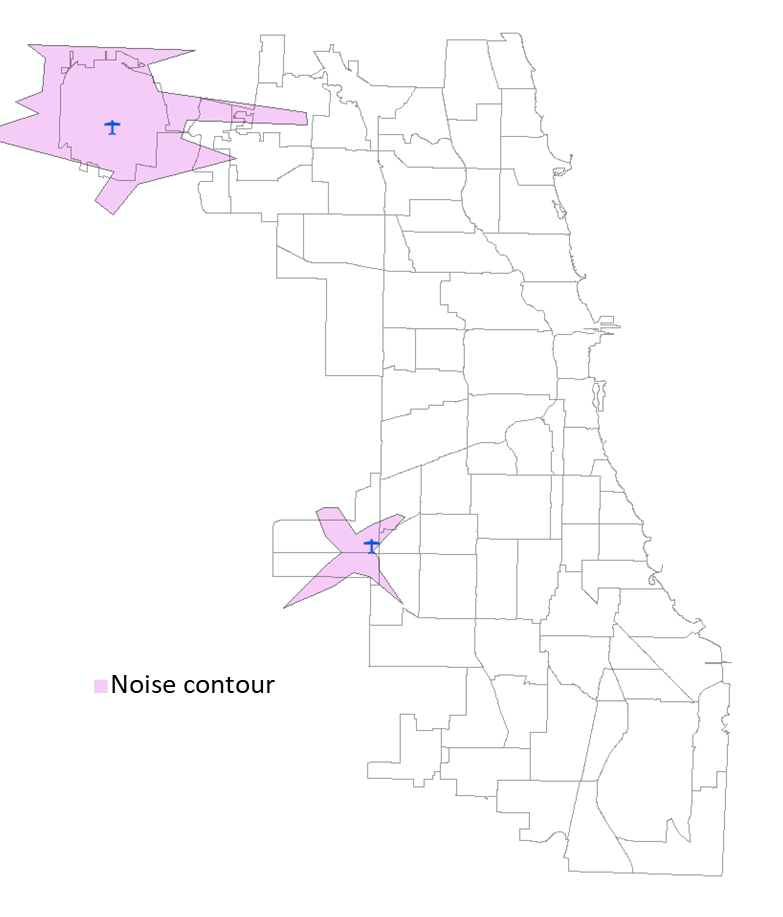


c) Ground traffic – Roads and Streets d) Ground traffic – Public Transit


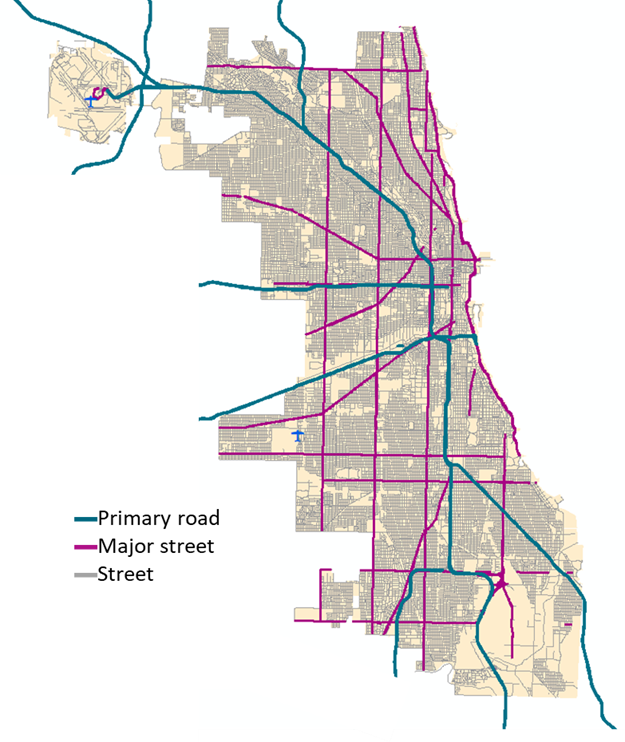

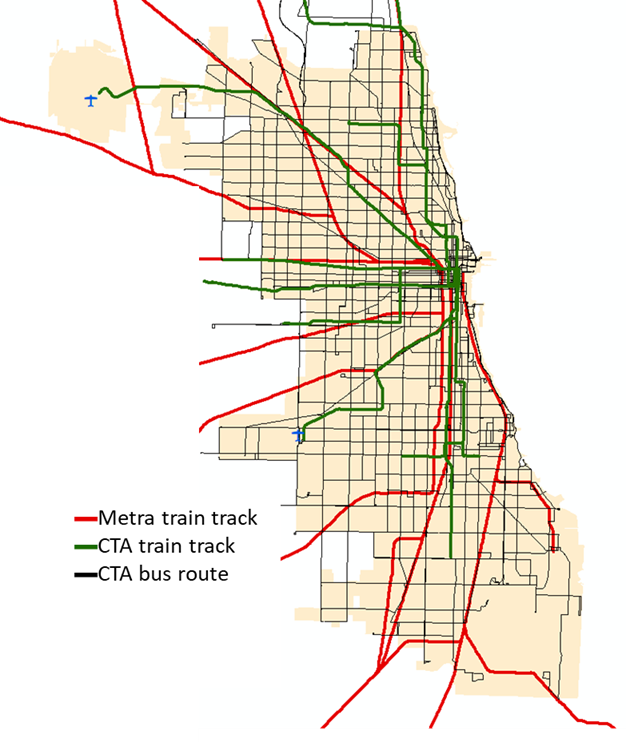


e) Natural environment – Land and Water f) Natural environment - Greenness


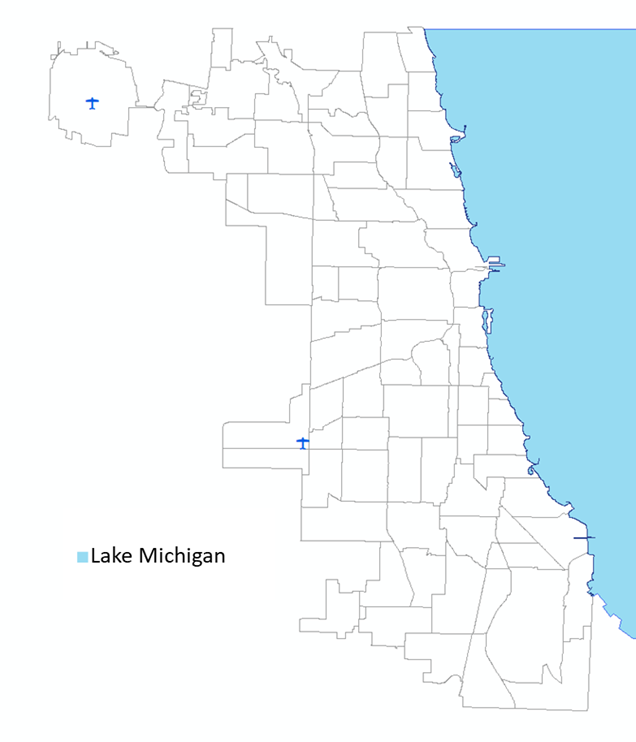

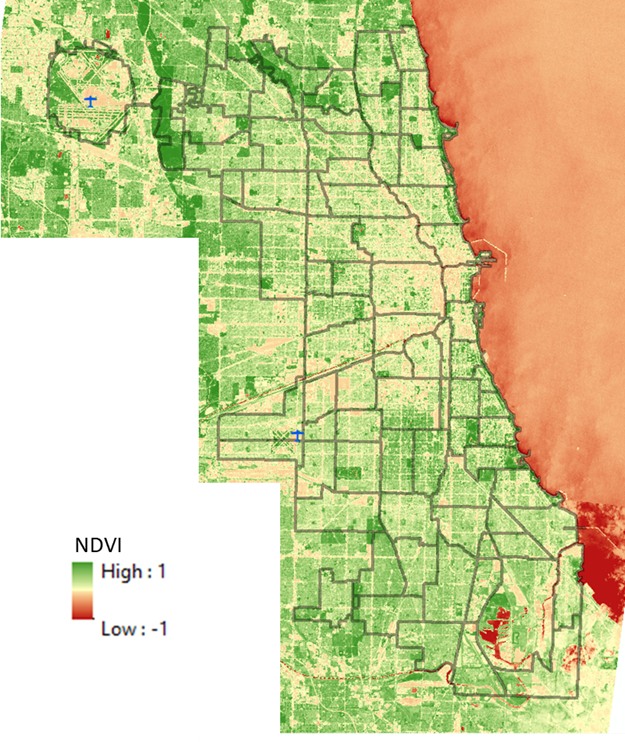


**S2 Figure. Noise sampling sites (black triangles) and areas where the LUR model will predict outside the range of geographically-based variables (D_CTA_, PRD_100_, NDVI_100_, and NDVI_500-100_) represented by the noise sampling sites (white).**


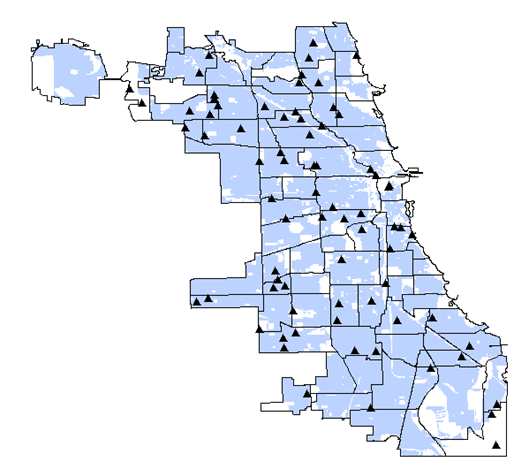


**S3 Figure. Regression diagnosis and the predicted noise levels for training and validation set.** (a) Scale-location plot shows linearity assumption is valid, but a slightly heteroscedasticity; (b) Q-Q plot shows good assumption of normality among standardized residuals; and (c) predicted noise levels are similar to the observed values in both the training and validation sets.


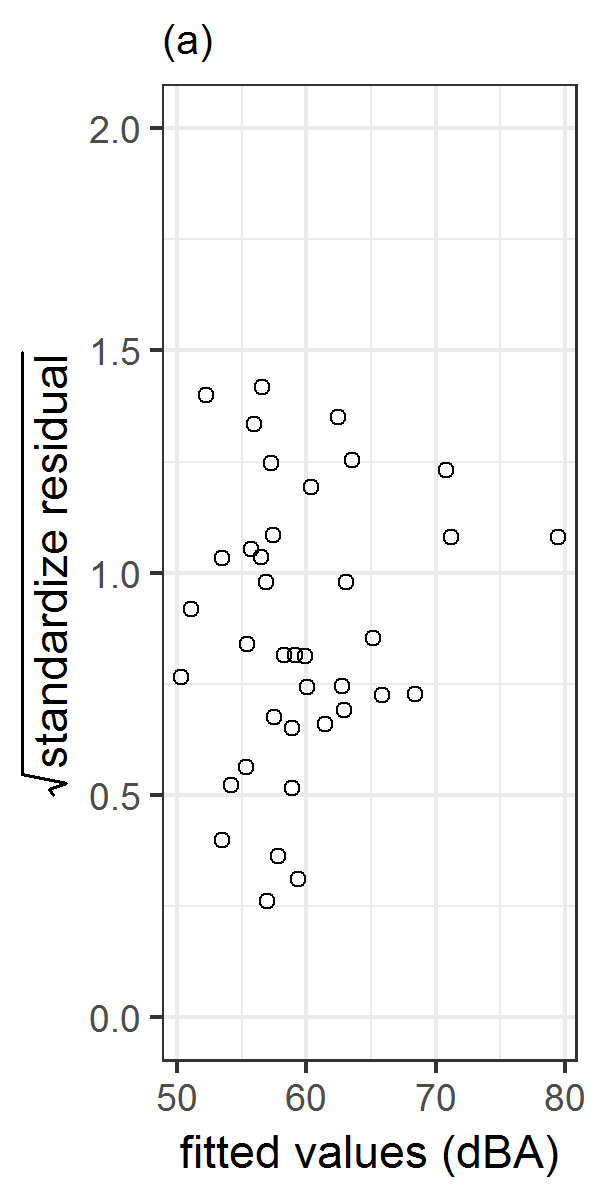

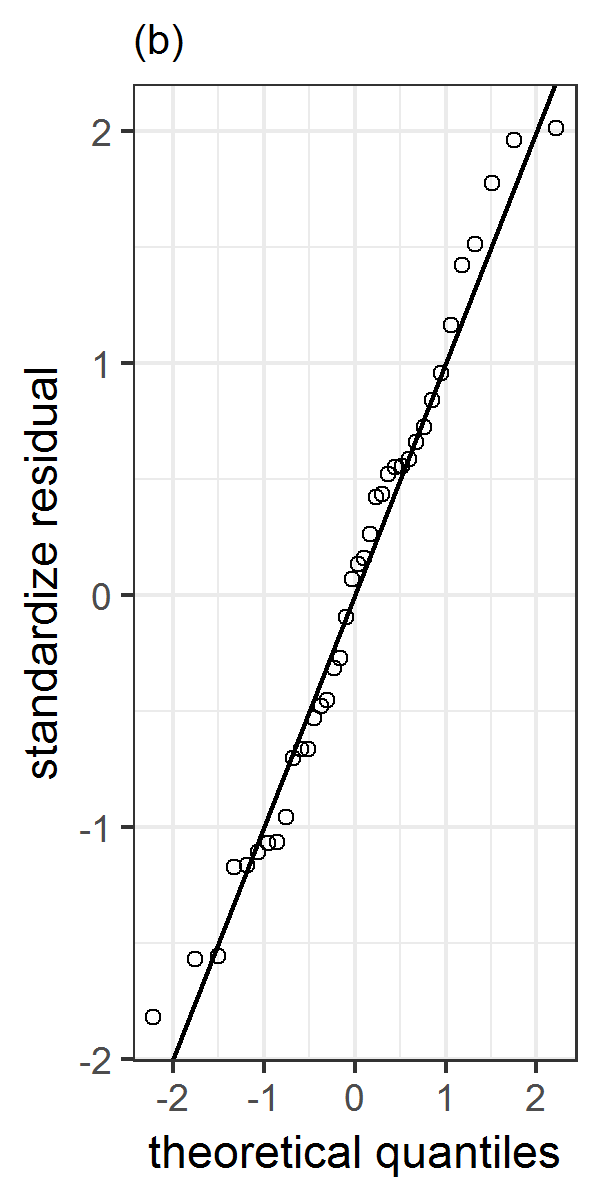

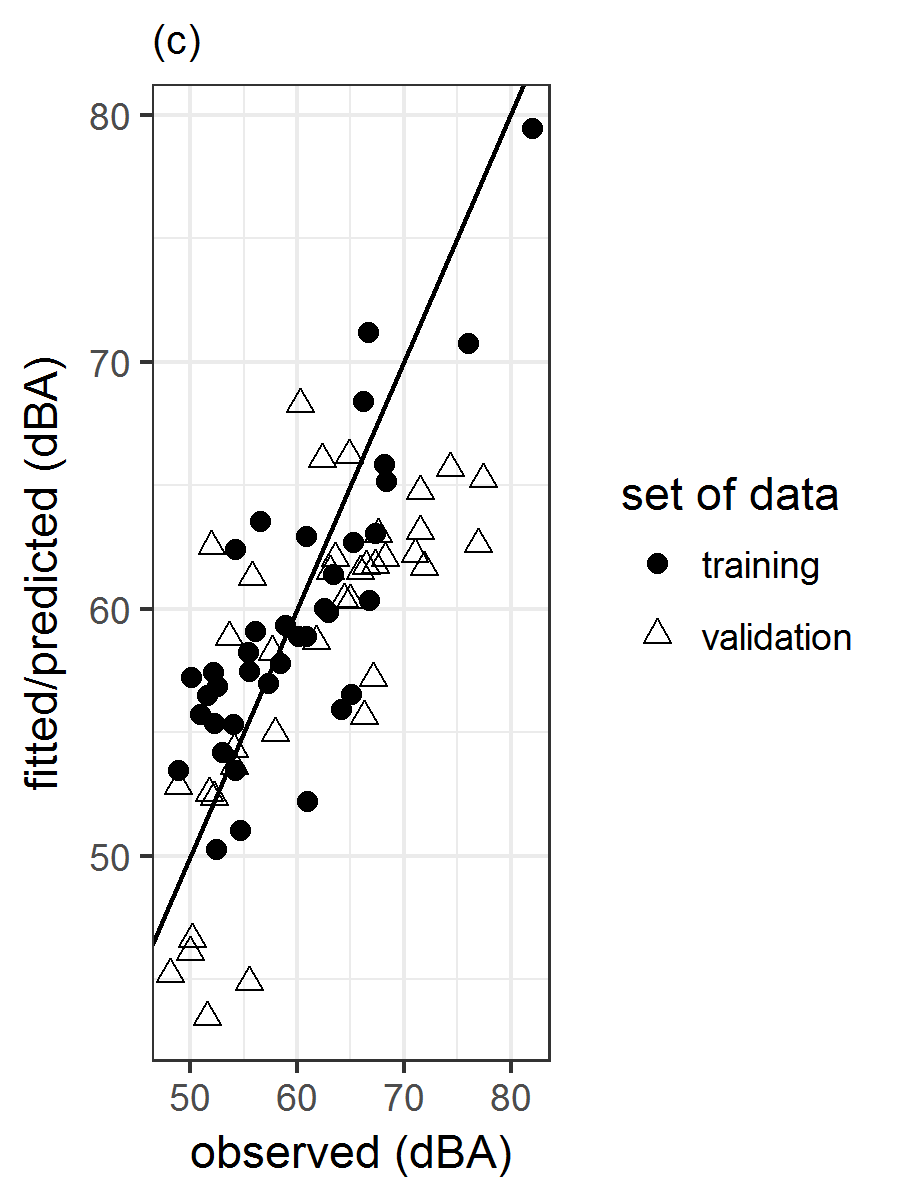


**S2 Table. Fitted land use regression model predicting noise (Leq_-5min_) in Chicago:** Sensitivity analysis excluding two locations close to Lake Michigan

| **Model** | | | | | **Training Set**  **(n=37)** | | **Validation Set**  **(n=36)** | |
| --- | --- | --- | --- | --- | --- | --- | --- | --- |
| **Variables** | **β** | **SE** | **p-value** | **VIF** | **adj-R^2^** | **RMSE**  **(dBA)** | **adj-R^2^** | **RMSE (dBA)** |
| Intercept | 72.61 | 3.00 | <.001 |  | 0.59 | 4.73 | 0.53 | 5.54 |
| D_CTA_ | -1.74×10^-3^ | 4.53×10^-4^ | <.001 | 1.21 |  |  |  |  |
| NDVI_100_ | -31.30 | 7.93 | <.001 | 1.88 |  |  |  |  |
| PRD_100_ | 2.65×10^-2^ | 8.67×10^-3^ | 0.004 | 1.39 |  |  |  |  |
| NDVI_500-100_ | -25.08 | 10.46 | 0.023 | 2.10 |  |  |  |  |

**S3 Table. Univariate spatial auto regression models of the association between community area daytime noise level and racial and ethnic demographics.**

| **Demographic Variable** | **Coefficients (Standard Error)** | | | |
| --- | --- | --- | --- | --- |
|  | **β** | **Quartile 2** | **Quartile 3** | **Quartile 4** |
| % White Population | 1.4 (3.89)  p = 0.007 | 0.94 (0.90)  p = 0.296 | 1.13 (0.90)  p = 0.209 | -0.03 (0.90)  p = 0.973 |
| % Black Population | 11.1 (3.97)  p = 0.005 | -0.17 (0.91)  p = 0.854 | -0.59 (0.91)  p = 0.518 | 0.03 (0.91)  p = 0.975 |
| % Asian Population | 12.5 (4.27)  p = 0.003 | -0.07 (0.92)  p = 0.938 | 0.18 (0.91)  p = 0.844 | 1.27 (0.94)  p = 0.176 |
| % Hispanic Population | 10.8 (3.84)  p = 0.005 | -0.93 (0.90)  p = 0.304 | -0.56 (0.92)  p = 0.541 | -0.457 (0.89)  p = 0.609 |

**S4 Table. Mean total Chicago Transit Authority (CTA) train track and highway length by income quartile in Chicago community areas.**

| Income quartiles | Total CTA Track (m) | Total Highway (m) |
| --- | --- | --- |
| 1 | 1551 | 5947 |
| 2 | 1433 | 8714 |
| 3 | 1186 | 4629 |
| 4 | 3252 | 5841 |

**S4 Figure. Distribution of mean total Chicago Transit Authority (CTA) train track length (left) and highway length (right) by income quartile in Chicago community areas.**


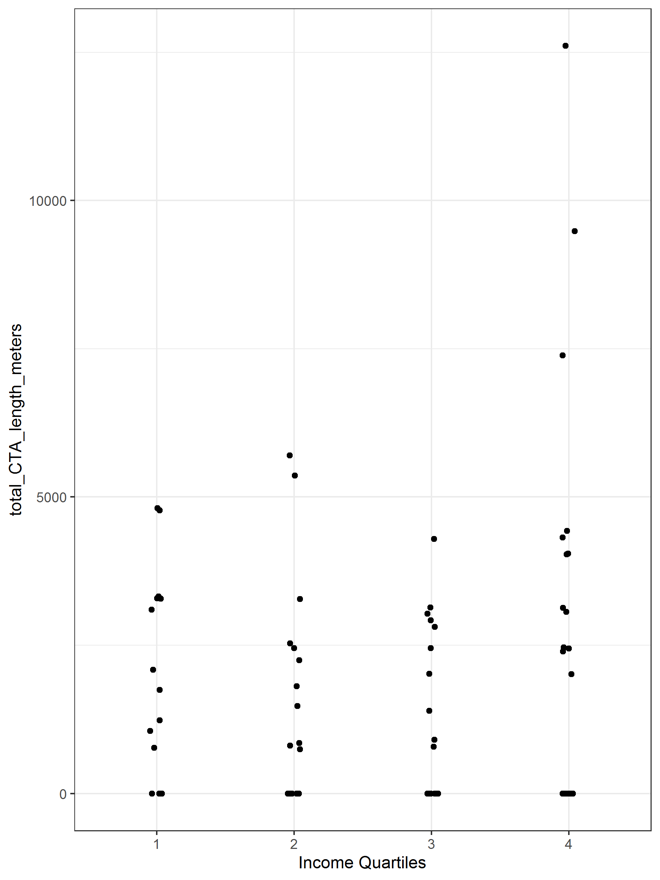

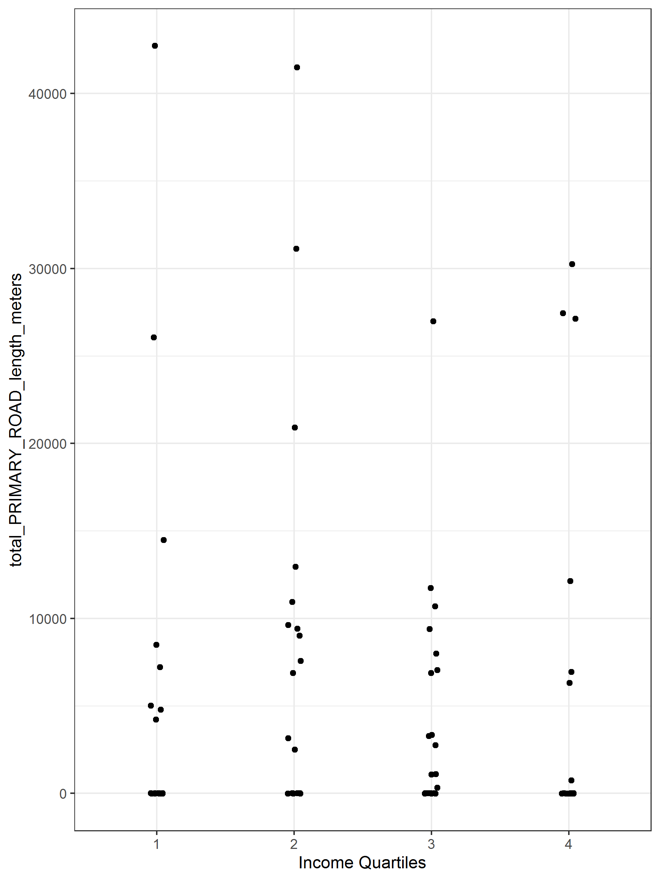

Supplement: S1 File — (DOCX) [file pone.0254762.s001.docx]
